# Customized 3D-Printed Scaffolds for Alveolar Ridge Augmentation: A Scoping Review of Workflows, Technology, and Materials

**DOI:** 10.3390/medicina61071269

**Published:** 2025-07-14

**Authors:** Saeed A. Elrefaei, Lucrezia Parma-Benfenati, Rana Dabaja, Paolo Nava, Hom-Lay Wang, Muhammad H. A. Saleh

**Affiliations:** 1 Department of Periodontics and Oral Medicine, School of Dentistry, University of Michigan, Ann Arbor, MI 48109, USA; elrefaei@umich.edu (S.A.E.); lucrezpb@umich.edu (L.P.-B.);; 2 Department of Mechanical Engineering, School of Engineering, University of Michigan, Ann Arbor, MI 48109, USA

**Keywords:** surgical mesh, bone regeneration (BR), 3D printing, 3D imaging, computer-assisted designs, computer-aided manufacturing, CAD/CAM, digital, customized

## Abstract

*Background and Objectives:* Bone regeneration (BR) is a cornerstone technique in reconstructive dental surgery, traditionally using either barrier membranes, titanium meshes, or perforated non-resorbable membranes to facilitate bone regeneration. Recent advancements in 3D technology, including CAD/CAM and additive manufacturing, have enabled the development of customized scaffolds tailored to patient needs, potentially overcoming the limitations of conventional methods. *Materials and Methods:* A scoping review was conducted according to the PRISMA guidelines. Electronic searches were performed in MEDLINE (PubMed), the Cochrane Library, Scopus, and Web of Science up to January 2025 to identify studies on digital technologies applied to bone augmentation. Eligible studies encompassed randomized controlled trials, cohort studies, case series, and case reports, all published in English. Data regarding digital workflows, software, materials, printing techniques, and sterilization methods were extracted from 23 studies published between 2015 and 2024. *Results*: The review highlights a diverse range of digital workflows, beginning with CBCT-based DICOM to STL conversion using software such as Mimics and Btk-3D^®^. Customized titanium meshes and other meshes like Poly Ether-Ether Ketone (PEEK) meshes were produced via techniques including direct metal laser sintering (DMLS), selective laser melting (SLM), and five-axis milling. Although titanium remained the predominant material, studies reported variations in mesh design, thickness, and sterilization protocols. The findings underscore that digital customization enhances surgical precision and efficiency in BR, with several studies demonstrating improved bone gain and reduced operative time compared to conventional approaches. *Conclusions*: This scoping review confirms that 3D techniques represent a promising advancement in BR. Customized digital workflows provide superior accuracy and support for BR procedures, yet variability in protocols and limited high-quality trials underscore the need for further clinical research to standardize techniques and validate long-term outcomes.

## 1. Introduction

Bone regeneration (BR) is a common surgical approach for bone reconstruction and augmentation. Guided bone regeneration (GBR) is based on using bone-grafting materials combined with barrier membranes, which allow tissue exclusion during the healing phase to promote bone regeneration and enable the placement of dental implants in anatomical areas with vertical, horizontal, or combined bone defects [1,2,3,4].

Wang and Boyapati described, in 2006, the four major biologic principles (the PASS principle) necessary for predictable bone regeneration and, between them, the importance of space maintenance/creation to facilitate adequate space for bone ingrowth, and none of them exclusively involved using barrier membranes [5,6]. The barrier function is one of the key elements in GBR since it protects against the penetration and colonization of soft tissues [7]. Since expanded polytetrafluoroethylene (e-PTFE) was first introduced in 1969 [8], and was later sintered with pores between 5 and 20 mm to provide some periosteal blood infusion, several other barriers were introduced, including high-density polytetrafluoroethylene (D-PTFE) and resorbable collagen and polymeric membranes. However, all showed mechanical hindrance due to the lack of space maintenance/creation function, as the membrane collapses into the underlying soft tissue defect, leading to subsequent shrinkage of the underlying graft [9]. This was later mitigated by using a titanium framework to provide space maintenance and prevent the dPTFE membrane collapse [10,11].

In their classic forms, commercially available titanium meshes were advocated as a gold standard for vertical and horizontal alveolar ridge augmentation (ARA) for their malleability and mechanical integrity, which maintains an unaltered graft space. However, these membranes pose challenges during surgery, including difficulties in modeling and fixing, which renders the process time-consuming. The perforated structure of the mesh does not impede the periosteal blood supply; however, it does not restrain fibrous tissue invasion, is difficult to remove surgically, and contributes to soft tissue dehiscence [12,13]. Van Steenberghe et al., in 2003, utilized occlusive titanium barriers to completely conceal the underlying graft and safeguard the blood clot while simultaneously utilizing the high endosteal perfusion of the maxillary and calvarial bones to guide bone regeneration [14].

The rapid advancement of 3D digital technology has seen the introduction of a customized 3D computer-aided design and manufacturing (CAD-CAM) workflow, resulting in the development of a CAD/CAM titanium mesh for protected bone augmentation [15]. Customized titanium meshes have gained popularity due to their ease of use, efficiency, and adaptability to the patient’s specific anatomy. These meshes are durable and rigid and provide excellent structural support for bone grafts, reducing the risk of graft displacement [16].

Fabricating customized titanium meshes using conventional subtractive manufacturing methods is highly challenging [4]. In this context, the layer-by-layer technique of additive manufacturing (AM) has emerged as a key technology for producing complex, patient-specific scaffolds and components for biomedical applications. Several AM techniques are employed for titanium printing, including powder bed fusion (PBF), direct energy deposition (DED), and binder jetting [17,18,19].

The PBF techniques selective laser melting (SLM) and electron beam melting (EBM) are widely used for metal 3D printing. More specifically, SLM uses a high-powered laser to selectively fuse metal powder according to a target design. SLM has the capability to fabricate intricate, high-resolution porous architectures with excellent mechanical properties, making it a suitable choice for patient-specific applications [20]. SLM is especially advantageous for fabricating porous titanium components because of its superior resolution, precision, and mechanical performance [21,22]. However, a limitation of the SLM process is the partially melted residual powders on the surface, requiring post-processing treatments such as chemical or electrochemical etching, followed by autoclave sterilization to ensure the component is free of biological contaminants [23,24].

Another advantage of SLM is the elimination of internal support structures, which reduces design constraints and allows for the production of high-resolution components with micro-scale features [25]. These advantages make SLM an optimal technique for creating scaffolds and meshes tailored to individual patient needs.

The design and analysis process using 3D scans of defective sites is a promising means to advance operative procedures as well as in fabricating implants, especially in severely defective sites. There are structured workflow algorithms that currently exist for this. According to a narrative review, the process for the 3D printing of patient-specific implants (including dental and grafting applications) consists of three main phases: image acquisition, post-processing, and 3D printing [26,27,28]. The image acquisition step involves obtaining patients data, typically through computed tomography scans. In the post-processing phase, the site is reconstructed according to the scan, and using computer-aided design (CAD), the implant or graft is designed. The resulting file from the CAD is then 3D-printed. The image acquisition step can either be used to provide a three-dimensional model of the defective site or be used as a guide to design the specific component. This process generally applies to all 3D-printed patient-specific components.

Kim et al. conducted a randomized study with a customized 3D-printed ceramic bone graft, where they used CAD software to design a patient-specific bone graft based on radiographic and computed tomography scans [27]. Similarly, Tallarico et al. [28] conducted a case study of a customized 3D-printed titanium mesh for an alveolar ridge because of sever. This process followed a three-step process: first, a computed tomography scan was acquired; STL files were then derived from the DICOM data for analysis; and the customized titanium mesh was designed using CAD/CAM software according to the virtual planning of the target area. The final titanium mesh was 3D-printed and obtained from New Ancorvis Srl. In another study, Ivanovski et al. used a similar framework to design and verify a scaffold over a contour of missing bone for a staged alveolar bone augmentation before implant placement [29]. A computed tomography scan was used to generate an STL file of the defect, which was then used to design the scaffold, and verified by segmenting the area to ensure an accurate fit. The final scaffold was fabricated using bioprinting.

Overall, the framework for fabricating patient-specific scaffolds consists of image-acquisition techniques to characterize the defective area or use as a guide for the CAD-based design process of the patient-specific structure. The resulting STL file is then fabricated using appropriate 3D printing methods.

In this scoping review, we systematically mapped the 3D planning and printing literature, focusing on current advancements and emerging trends. The review provides clinicians with a comprehensive overview of the state of the art, including the latest developments in software, materials, printing techniques, and sterilization methods.

## 2. Materials and Methods

This scoping review adhered to the Preferred Reporting Items for Systematic Reviews guidelines and Meta-Analyses extension for Scoping Reviews (PRISMA-ScR). Ethical approval is not required for this scoping review.

### 2.1. PICO Question

In adult patients undergoing bone regeneration (BR) procedures, what is the impact of using customized digital workflows for 3D planning and printing on clinical and radiographic outcomes compared to conventional methods?

- P (Population): Adult patients requiring BR for alveolar ridge augmentation.
- I (Intervention): To explicitly include both additive (printing) and subtractive (milling) manufacturing of scaffolds.
- C (Comparison): Conventional BR techniques using preformed or manually shaped barrier membranes and grafts.
- O (Outcomes): Clinical outcomes (e.g., implant success, incidence of complications, operative time) and radiographic outcomes (e.g., residual bone height, immediate and final bone gain).

The search strategy used in all the mentioned databases was the following: (cad cam[Title/Abstract]) OR (customized[Title/Abstract]) OR (computer aided[Title/Abstract]) OR (digital[Title/Abstract]) OR (digitally[Title/Abstract]) OR (digital workflow[Ti-tle/Abstract]) OR (digital planning[Title/Abstract]) AND (bone reconstruction[Title/Abstract]) OR (bone regeneration[Title/Abstract]) OR (bone grafting[Title/Abstract]) OR (alveolar ridge augmentation[Title/Abstract]).

### 2.2. Article Eligibility Criteria

We included peer-reviewed studies such as randomized controlled trials, non-randomized trials, cohort studies, case series, and retrospective studies that evaluated digital workflows for BR. Eligible studies were required to report at least one clinical or radiographic outcome and be published in English between 1 January 2015 and 1 January 2025. In vitro studies, animal studies, commentaries, narrative reviews, unpublished data, expert opinions, and isolated case reports were excluded.

### 2.3. Information Sources and Search Protocol

An electronic search was conducted in MEDLINE (PubMed), the Cochrane Library, Scopus, and Web of Science. Comprehensive search strategies were formulated using relevant MeSH terms, keywords, and free-text terms focused on digital planning, 3D printing, and BR. The final search was executed on 27 February 2025.

**Figure 1:**
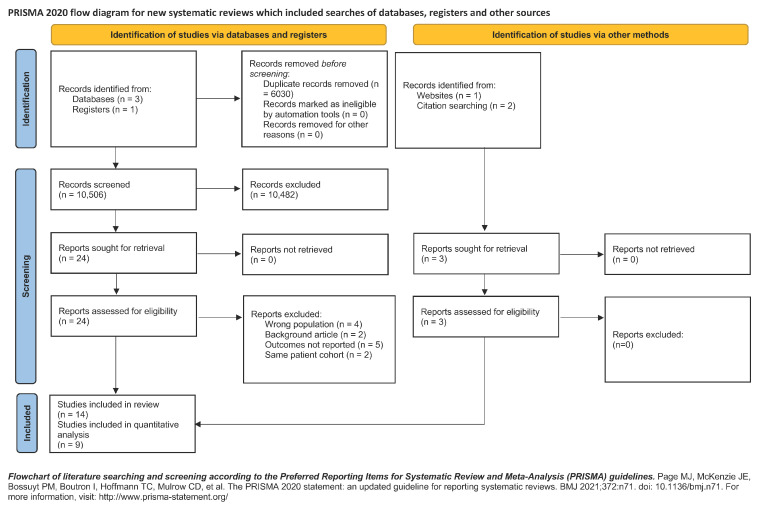
Illustrates the PRISMA flow diagram.

A PRISMA flow diagram is included (Figure 1), detailing the number of records identified, screened, and included in the review, along with reasons for exclusion at each stage.

### 2.4. Article Selection

Two independent reviewers (SE and LPB) screened the titles and abstracts using the Covidence Systematic Review Software (Veritas Health Innovation, Melbourne, Australia). Full-text articles were then assessed against the pre-established inclusion and exclusion criteria. Discrepancies were resolved through discussion, with a third reviewer (MHAS) serving as an arbiter if consensus was not reached.

### 2.5. Data Extraction

Data were independently extracted by two reviewers (SE and LPB) and subsequently verified for accuracy. The extracted information included study characteristics (e.g., publication year, design, sample size, follow-up duration), details of the digital workflow (e.g., planning software, file conversion methods, design protocols), materials used, printing and sterilization techniques, and the reported clinical and radiographic outcomes. When multiple measurements per site were reported, their average was calculated and used for analysis.

## 3. Results

This literature review analyzes findings from 23 studies, summarized in Table 1. The studies included in this review were published between 2015 and 2024. Specifically, the review encompasses nine randomized clinical trials [27,30,31,32,33,34,35,36,37], one prospective study [38], two pilot studies [39,40], five case series [41,42,43,44,45], five retrospective case studies [16,46,47,48,49], and one clinical trial [50].

### 3.1. Workflow (Here We Explain the Common Workflow of All Different Scaffolds (Figure 2))

**Figure 2:**
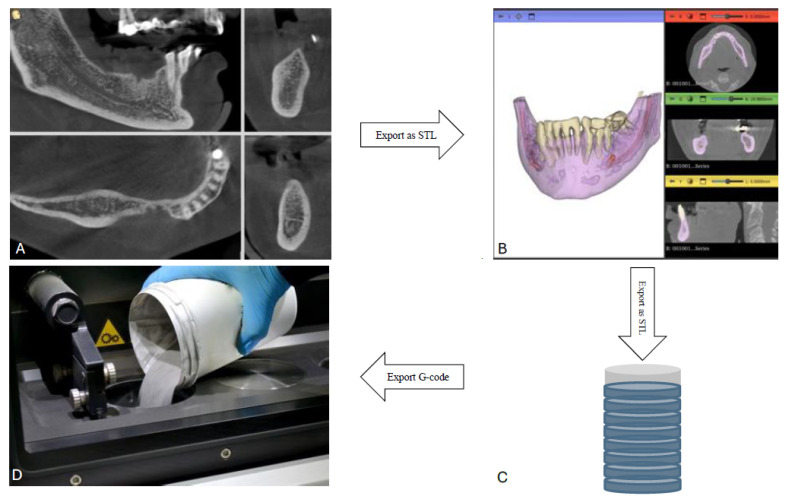
Represents the steps of custom mesh printing: (**A**) The patient data available in DICOM file extension. (**B**) Converting the patient data into STL extension. (**C**) The preprinting parameters of the customized mesh (slicing, layer thickness, and powder material parameters (laser power and speed)). (**D**) Adding the metal powder to the metal reservoir of the 3D printing machine.

**Figure 3:**
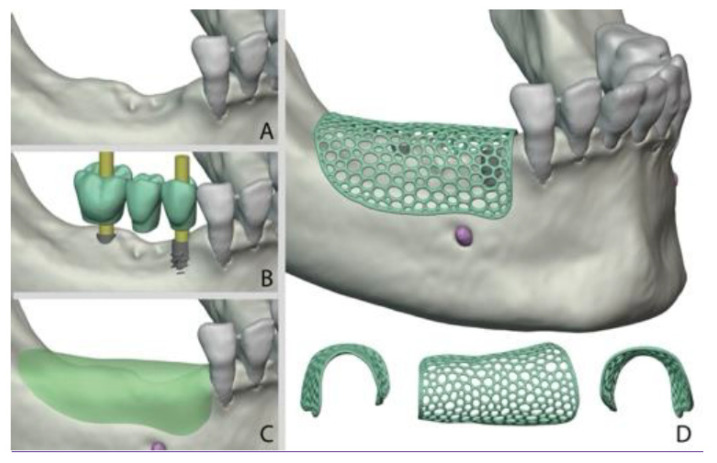
Represents the step of designing the customized mesh: (**A**) The initial situation with the defective bone. (**B**) The proposed implant placement in the defected ridge. (**C**) The defective ridge was augmented to estimate the required volume of augmentation. (**D**) The final design of the customized mesh to accommodate the required augmentation volume with the required design.

- The first step is to analyze the patient bone condition using cone beam computed tomography (CBCT), which provides the surgeon with a converted Digital Imaging and Communications in Medicine (DICOM) file format (Figure 2A).
- The second step is to transfer these DICOM files to a Standard Triangulation Language (STL)-extension file that can be read that are analyzed using 3D software. Different software could be used, like the Dragonfly software (Figure 2B).
- With the patient data available on the 3D software, the surgeon can augment the defective areas and have a better visualization of what the proposed treatment plan would end up with (Figure 3).
- Then, after augmenting the areas with the desired amount of bone (Figure 3C), the membrane can be designed to correspond to those augmented areas. The thickness of the membrane and design (perforated on non-perforated), number and locations of fixation screws, and implant location opening can be designed in the frame (Figure 3D).
- The preprinting parameters of the desired membrane are predetermined before printing (Figure 3C).
- After the design is confirmed, a 3D printer can then be used to print the desired customized mesh (Figure 2D).

The flowchart indicates that the second step in the 3D printing process involves using a secondary software application to convert STL files into a format that the printer can read. The specific software utilized varies with the manufacturer of the printing machine; however, results differ across studies unless they are authored by the same research team [34,35,39]. In some studies, the printer manufacturer and associated software are not specified [31,33,38,40,41] (Table 2).

### 3.2. Digital Planning Software

The first step in planning a 3D mesh involves converting Digital Imaging and Communications in Medicine (DICOM) files from a cone beam computed tomography (CBCT) scan into Standard Triangulation Language (STL) files. This conversion allows for accurate planning of the necessary augmentation of hard and soft tissues based on the final prosthetic objectives.

Six of the nineteen studies employed the same software—Mimics software (Materialise, Leuven, Belgium)—though reported as different versions based on updates from the respective year of the study [27,31,33,37,38,40,41,46,49]. Five studies used the Biotec software Btk-3D^Ⓡ^ (Biotec Srl, Dueville, Vicenza, Italy), with four of these studies having the same principal investigator [32,35,36,39]. Felice et al. (2024) [30] utilized the 3D Slicer software (Boston, MA, USA), Sagheb et al. [47] and Chiapasco et al. [16] referred to using Yxoss CBR^®^ backward by ReOss, Li S. et al. [48] mentioned the TRIOS 3 (3Shape, Copenhagen, Denmark) for their conversion, while Meckha et al. (2023) [45] used Dent-Plan (NECTEC, Thailand). The four case series did not specify the name of the software used [42,43,44,50].

### 3.3. Materials Utilized

Regarding materials, sixteen studies reported using titanium alloy. Of these, three specified the use of grade IV titanium [16,41,42,43], with Mohamed et al. [41] noting it was supplied as a block. Four studies used a different grade of titanium (Ti64) in the form of a pre-alloyed Ti_6_AlV_4_ fine powder [38,40,46,49], while three studies did not specify the titanium type [30,32,36,47,48,50]. In the studies by Cucchi et al. [34,35,39], grade V titanium was used in micro-powder form.

Following the same digital workflow, it is possible to produce a mesh in other materials, such as Poly Ether-Ether Ketone (PEEK). The randomized clinical trial by El Morsy et al. [31] described the use of a mesh made from a PEEK block, whereas another study [33] compared a test group using custom PEEK meshes to a control group with 3D-printed titanium meshes.

Using STL models and DICOM files, it is possible to obtain 3D bone models on which a simulation of the bone augmentation can be performed using dedicated computer-aided design (CAD) software to create it or sintering 3D bone graft materials.

Three studies focused on the development of this new technological innovation. Kim et al. [27] printed a synthetic alloplastic bone graft using a 60:40 mixture of hydroxyapatite and β-tricalcium phosphate. Mekcha et al. [45] utilized 3D-printed nanohydroxyapatite in their case series, while Wang et al. [37] milled pre-cut cortico-cancellous freeze-dried allogenic bone blocks for their clinical trial.

### 3.4. Material Thickness

Measurements concerning the dimensions or thickness of the bone graft materials cannot be provided, as they are strictly related to the size of the defects they aim to fill or regenerate. However, a customized membrane should have sufficient stiffness to create and maintain a suitable space for the intended BR, which is mainly related to the thickness of the membrane. The thickness of the meshes reported in the studies varies in order to meet the clinician’s exigencies and to exert external force pressure, ranging from 0.1 mm (initially calibrated at 0.3 mm and reduced through metal lamination after printing) [38] to 2 mm [33]. The average thickness across studies was 0.5 mm, but seven studies did not specify this measurement [16,31,32,42,43,47,48].

### 3.5. Type of Mesh

Concerning the type of mesh, they are categorized in the tables as perforated, semi-occlusive, or occlusive/not perforated. Three studies used occlusive titanium mesh [30,41,44], and one study employed an occlusive PEEK mesh [31]. A randomized controlled trial by Felice et al. [30] compared an occlusive mesh with a semi-occlusive one that had pores measuring 0.3 mm in diameter. As shown in the table, most of the customized titanium mesh had a design with pores: sixteen studies utilized perforated titanium mesh [16,32,33,34,35,36,38,39,40,42,43,46,47,48,49,50]. The RCT from Mounir et al. [33] contrasted a control group with perforated titanium mesh against a test group that used perforated custom PEEK mesh. Another aspect that has been cited in the same column is the pore size. The pores of barrier membranes are channels of nutrient and oxygen exchange and are closely related to the blocking ability of cells. Only two studies specified the pore size, calibrated at 1 mm in diameter for both clinical trials [38,50].

### 3.6. Printing Method

In terms of printing methods, the studies can be categorized into three distinct types: direct metal laser sintering (DMLS) [16,27,32,36,38,40,42,45,51], five-axis milling [31,33,37,41,44], and selective laser melting (SLM) [34,35,39,46,47,49,50]. Table 3 discusses the different methods for custom mesh fabrication, with the advantages and disadvantages of each technique. Only the retrospective clinical study by Li S. et al. [48] does not specify the printing method and its 3D printer. Milling is a machining process that removes material from a solid piece through cutting with a rotating tool. In contrast, sintering involves fusing powder particles by applying heat and pressure below their melting point, creating a solid mass without physically removing the material. Essentially, milling is a subtractive process, while sintering is an additive process in manufacturing.

Additionally, the study by Ciocca et al. [38] used direct metal laser sintering (DMLS) with titanium alloy in fine powder form rather than as a block. Conversely, studies by Cucchi et al. [34,35,39] indicate that selective laser melting was used for titanium alloy grade V in micro-powder form.

The SLM process mainly consists of a powder bed, build plate, recoater, and laser source. Prior to printing, the 3D bone scan or CAD model is imported into preprocessing software to generate an input file that defines the print parameters, part orientation, and quantities, all of which can influence the print quality [50]. Printing occurs in an inert atmosphere to prevent oxidation [23]. The process begins with a fresh layer of powder recoated onto the build plate, which is then selectively fused together by a high-energy laser in a pattern according to the CAD model. Following the completion of each layer, the build plate is incrementally lowered by the thickness of a single layer before a new layer of powder is deposited. This iterative process continues until the final part is fabricated (Figure 2D).

### 3.7. Sterilization Method

Regarding sterilization methods, three articles noted that titanium meshes were autoclaved at 135 °C for 15–20 min [33,36,44]; while three studies indicated that the meshes were sterilized using a 2.4% glutaraldehyde solution for 12 h [31,33,41,47,48]. Even though the retrospective study by Li et al. [49] does not specify the use of an autoclave, it reports a sterilization method by high temperature and high pressure. The remaining studies either received the meshes already sterilized from the manufacturer or did not specify a sterilization method. As for the 3D bone materials, only one study mentioned sterilization through soaking in a disodium hydrogen phosphate solution for 24 h to convert it into a hydroxyapatite structure before drying and sterilizing with ethylene oxide gas [45].

### 3.8. Fitting/Fixation

All clinical trials during the surgical phase reported using a limited number of fixation screws, ranging from 2 to 3, primarily on the buccal side. Titanium pins were utilized to secure freeze-dried allogenic bone [37], while in the other two studies involving 3D bone graft material, no fixation screws were required because the materials fit perfectly into the defects without any movement [27,45].

## 4. Discussion

The rise in biomaterial engineering and CAD-CAM technology has resulted in the creation of new alternatives to autograft materials, such as customized meshes that can be tailored to meet the specific needs of clinicians and the requirements of various surgical procedures. Our research aimed to investigate the workflow, techniques, and materials used for 3D-printed scaffolds used for BR techniques.

Due to the novelty of this topic, the studies included in this review were published within the last ten years. Two other recent reviews in the literature [52,53] agreed with the authors’ opinion that partially or fully digital workflows can be successfully used in BR and advanced regenerative surgeries. The review by Shi et al. [52] limited their analysis to customized barrier membranes, describing in detail the workflow, the materials, advantages, and disadvantages of using them for BR techniques; the other narrative review, by Tommasato et al. [53], aimed to explore the application of digital technologies for bone augmentation procedures, proposing a decision tree on the digital workflow evaluating hard and soft tissue defects and the appropriate surgical techniques.

The innovation of this paper is to explore, report, and explain to clinicians the several steps necessary to perform this digital workflow, including the software, materials, and 3D printer cited in the literature.

The initial step in most workflows involved converting and transforming DICOM data into STL files. Although all studies except three [42,43,44] report the software used for this process, we believe that this detail is crucial for accurately reporting the methods of the clinical trials. The most commonly used software for DICOM extraction and transformation into STL files is Mimics (Materialise, Leuven, Belgium), followed by Btk-3D^®^ (Biotec Srl, Dueville, Vicenza, Italy).

The second step of the workflow often involves a second software that is used to design the mesh/bone according to clinician/surgery necessity. This step could be avoided in the case of CAD software directly related to the CAM 3D printer provided by the company. Hence, as reported in several studies, the STL files are directly sent to the company that will design, print, and sometimes sterilize the mesh, always under the clinician’s final approval [16,32,34,35,36,39,42,43,47]. The evaluation also applies to bone graft material. According to Wang et al. [37], there were no details reported about the design software used. We only know that after the STL files were sent, the company milled, cleaned, and packaged freeze-dried allogenic bone from the iliac crest for surgical use.

Another comment regarding this type of software and its advantages is related to the possibility of evaluating and comparing bone augmentation and the results obtained after the BR technique. In Yang et al. [46], a superimposition technique was used to compare the difference between the planned and created bone augmentation in bone defects using the same software (3-Matic Materialise, Leuven, Belgium). A superimposition between the planned CAD model and that post-BR was performed [46]. Other studies used STL files pre- and post-surgery with different follow-ups to take their measurements and evaluate the success of the bone regeneration [27,34,38,39,45].

Some remarkable considerations are necessary regarding the material utilized. Even though the majority of the studies reported in the literature refer to titanium mesh, another type of barrier has been investigated in this study: Poly Ether-Ether Ketone (PEEK) [31,33]. El Morsy et al. [31] collected a case series involving fourteen patients with severely atrophied anterior maxillary alveolar ridges who underwent rehabilitation using custom-made CAD/CAM PEEK sheets as a containment system for an interposition mix of particulate autogenous and xenogeneic bone grafts. After conducting radiographic assessments, the authors concluded that the regenerative technique using patient-specific PEEK sheets successfully restored the deficient ridge [31]. Although this study lacked a comparative control group, one year earlier, Mounir et al. [33] published an RCT involving sixteen patients with vertically and horizontally deficient maxillary alveolar ridges. The participants were equally divided into two groups: one received a mix of particulate autogenous and xenogenic bone grafts loaded in a prebent titanium mesh (control group), while the other had patient-specific PEEK meshes applied (study group). The percentage of 3D bone gain in each group was compared, along with measurements of linear changes in the vertical and horizontal dimensions. The authors concluded that both techniques could be used as successful methods of ridge augmentation with no statistically significant differences between them [33]. The literary review by Shi et al. [52] suggests that the PEEK-based barrier has excellent mechanical properties, with an elastic modulus very similar to that of bone and great biocompatibility. Although customized PEEK meshes can reduce the operative duration to a certain extent compared with manually bent Ti meshes, they are more costly and are associated with an increased number of complications, such as infection.

Three studies analyzed the creation of 3D-printed bone. The randomized study by Kim et al. [27] evaluated the clinical effectiveness of a customized 3D-printed alloplastic bone material. Sixty patients requiring guided bone regeneration for implant installation following tooth extraction were randomly allocated into two groups. Half of the participants received a 3D-printed patient-customized bone graft material, while the other half received conventional block bone graft material. After five months, histological and radiological assessments showed significantly higher percent bone volume and a smaller tissue surface area in the 3D-printed group compared to the control group. However, no other significant differences were noted between the two groups. The study suggested that this material could serve as an alternative to conventional options [27]. These findings support a case series reported by Mekcha et al. [45], in which a specific block graft was successful in 10 out of 12 patients without requiring any graft adjustments.

Even though the results from these two studies appear promising, clinicians must evaluate the cost-effectiveness of the entire process. By highlighting only these two studies, it may seem there are no significant differences in bone gain, and the only advantages lie in reduced patient discomfort and a shorter fixation/adaptation time.

Wang et al. [37] compared the use of customized allogeneic bone blocks versus autogenous bone blocks. Although the sample size was limited to twenty-four patients, the study’s robust methodology revealed that the customized group resulted in greater horizontal bone gain and less horizontal bone resorption (measured at 1 mm below the alveolar ridge crest) at six months post-surgery compared to the autogenous bone block group. Additionally, the customized approach reduced the operative time in treating ridge augmentation [37]. These findings align with the existing literature, indicating satisfactory results when using CAD/CAM-customized titanium meshes for bone augmentation [54,55,56,57].

While most of the articles reviewed did not specify their sterilization techniques, it should be noted that titanium mesh is compatible with both hot and cold sterilization methods. In contrast, PEEK meshes can only be sterilized using cold methods. The situation varies for bone graft materials, as only one study reported using 3D-printed nanohydroxyapatite that was soaked in a disodium hydrogen phosphate solution for 24 h, then dried and sterilized with ethylene oxide gas [45]. Unfortunately, there is no information regarding the sterilization methods used in the other two studies.

This literature review focused on the 3D printing workflow process; therefore, no data or analysis comparing bone graft materials has been collected or reported in the tables. However, this could suggest an avenue for further clinical trials beyond just a systematic review and meta-analysis of this innovative topic.

There has been a growing interest in bioprinting, a layer-by-layer manufacturing technique that holds the capability to use organic materials to fabricate human tissue for bone regeneration. Bioprinting has the capacity to print scaffold constructs using synthetic or natural biomaterials, which can be either resorbable or non-resorbable depending on their composition. Natural biomaterials are biodegradable and often extracted from natural resources, either already existing in the human body or derived from polysaccharide biomaterials [58], include bioinks, polymer hydrogels, and collagen [58,59,60]. They exhibit mimicking characteristics of soft tissues. Although natural biomaterials, such as collagen and gelatin, exhibit excellent biocompatibility and cellular function, they lack sufficient mechanical strength. Thus, the combination of a hybrid material of a natural material printed on a synthetic scaffold is a promising technique to facilitate biological cues and cell viability while maintaining mechanical integrity [60].

## 5. Conclusions

Technological progress is moving in parallel with innovation in surgical techniques, supporting the clinician in all the phases: diagnosis, treatment plan, and surgery procedures. It can be considered helpful in patient–clinician communication, implant position planning, and during the follow-up period to evaluate bone gain as well. However, the number of studies (in particular RCTs) focused on the use of digital workflow is still limited. Also, more studies or clinical trials comparing 3D-customized bone and BR techniques and their advantages and complications are required.

The use of a titanium/SLM membrane was dominant, with better outcomes compared to other types of customized mesh fabrication.

## Figures and Tables

**Table 1:** The table summarizes the software, the material, the printing, and the sterilization method used by the authors to design, print, and use the 3D mesh or 3D bone graft. The last column of the tables includes the commercial companies mentioned by the authors.

Author and Year	Study Design	Software DICOM to STL	Software Mesh Design/Printer	Material	Thickness	Type of Mesh	Printing Method	Sterilization Method	Fitting/Fixation	Commercial Company
Mohamed et al. 2023	Case series	(Mimics Medical 19.0, Materialise) Belgium	Exocad software (DentalCAD) Germany	Titanium alloy grade IV blocks	0.7 mm	Occlusive titanium foil	Five-axis milling machine (CORiTEC 250i, imes-icore)	2.4% glutaraldehyde solution (Cidex, Johnson & Johnson) for 12 h, followed by steam sterilization	Fixed with screws	NA
Felice et al. 2024	RCT	3D Slicer software (Boston, MA, USA)	Meshmixer (Autodesk, San Francisco, CA, USA)	Titanium, without specifying	0.4 mm	- Semi-occlusive (0.3 mm diameter pores) - Occlusive titanium foil	NA	NA	Only vestibular fixation screws	3Dific, Perugia, Italy
El Morsy et al. 2020	RCT	Mimics 19.0; Mimics Medical 19.0 (Materialise, Leuven, Belgium)	Exocad software (DentalCAD, Germany)	Medical-grade PEEK blocks		Non-perforated PEEK	Five-axis milling machine	2.4% glutaraldehyde (Cidex, Johnson & Johnson co.) For 12 h.	Fixed with screws	NA
Giragosyan et al. 2024	RCT	Biotec Srl, Vicenza Italy	NA	Titanium without specifying	NA	Ti mesh with pores >2 mm	Selective laser sintering	NA	Fixed with titanium screws	Biotec Srl, Vicenza Italy
Mounir et al. 2019	RCT	Mimics 19 (Materialise, NV, Belgium)	Exocad software (DentalCAD, Germany)	Control group: prebent titanium mesh. Test group: PEEK.	Control group: NA. Test group: 2 mm.	Control group: perforated Ti mesh. Test group: perforated custom PEEK mesh.	Control group: (envisiontec GmbH, Gladbeck, Germany) to fabricate the virtually grafted 3D stereolithographic model that was then used as a guide for pre-bending of a readymade titanium mesh to create a space for the particulate graft intraoperatively. Study group: five axis milling machine from medical-grade PEEK.	Control group: autoclave. Test group: 2.4% glutaraldehyde (Cidex, Johnson & Johnson co.) For 20 min.	Both groups needed fixation with titanium screws. Collagen membrane (bio-gide, Geistlich Pharma, Switzerland) was used on both groups.	NA
Ciocca et al. 2018	Open prospective study	Mimic Innovation Suite software version 17.0 (Materialise, Leuven, Belgium)	CAD software (Freeform Modelling Plus, version 13.0, 3D Systems, Rock Hill, SC, USA)	Ti64 (a pre-alloyed Ti_6_AIV_4_ alloy in fine powder)	Calibrated at 0.3 mm, reduced to 0.1 mm by laminating the metal after printing	Holes in the mesh calibrated at 1 mm diameter	EOSINT M270 (Electro Optical Systems, Munich, Germany), a DMLS (direct metal laser sintering) machine	NA	1–2 osteosynthesis screws	NA
Cucchi et al. 2020	Pilot study	Btk-3D^®^, Biotec Srl, Dueville, Vicenza, Italy	CAD software (PLASTYCAD^®^, 3D COAT, Kiev, Ukraine)	Titanium grade 5 micro-powders, with layer size of 30 µm	Less than 0.5 mm	Perforated texture with calibrated holes	Selective laser melting (SLM) (50 W fibre laser with a wavelength of 1070 nm) (ProX-DMP100^®^, 3D system, Rock Hill, SC, USA)	Superficially polished, decontaminated in an automatic ultrasonic machine, packaged in a cleanroom under a controlled atmosphere, and sent for sterilization and clinical application	2–3 titanium screws	3D-Mesh^®^, BTK, Biotec Srl, Dueville, Italy
Cucchi et al. 2021	RCT	Btk-3D^®^, Biotec Srl, Dueville, Vicenza, Italy	CAD software (PLASTYCAD^®^, 3D COAT, Kiev, Ukraine)	Titanium grade 5 micro-powders, with layer size of 30 µm	Less than 0.5 mm	Perforated texture with calibrated holes	Selective laser melting (SLM) (50 W fibre laser with a wavelength of 1070 nm) (ProX-DMP100^®^, 3D system, Rock Hill, SC, USA)	Superficially polished, decontaminated in an automatic ultrasonic machine, packaged in a cleanroom under a controlled atmosphere, and sent for sterilization and clinical application	2–3 titanium screws	3D-Mesh^®^, BTK, Biotec Srl, Dueville, Italy
Cucchi et al. 2024	A non-inferiority RCT	Btk-3D^®^, Biotec Srl, Dueville, Vicenza, Italy	CAD software (PLASTYCAD^®^, 3D COAT, Kiev, Ukraine)	Titanium grade 5 micro-powders, with layer size of 30 µm	Less than 0.5 mm	Perforated texture with calibrated holes	Selective laser melting (SLM) (50 W fibre laser with a wavelength of 1070 nm) (ProX-DMP100^®^, 3D system, Rock Hill, SC, USA)	Superficially polished, decontaminated in an automatic ultrasonic machine, packaged in a cleanroom under a controlled atmosphere, and sent for sterilization and clinical application	2–3 titanium screws	3D-Mesh^®^, BTK, Biotec Srl, Dueville, Italy
Cucchi et al. 2024	A non-inferiority RCT	Btk-3D^®^, Biotec Srl, Dueville, Vicenza, Italy	CAD/CAM manufacturer (MyReoss, ReOss) Germany	Titanium, type not specified	NA	Perforated without specifying calibration	Laser sintering CAD/CAM technology	Placed directly in the autoclave and sterilized at 135 °C for 20 min in confined water vapor	3–4 osteosynthesis screws and/or titanium tacks	Yxoss CBR^®^, ReOss, (Filderstadt, Germany)
Yang et al. 2022	Retrospective case study	Mimics (Materialise, Leuven, Belgium)	3-Matic software (Materialise, Leuven, Belgium)	Titanium alloy powder (Dentarum Ti_6_Al_4_V, Germany)	0.3 mm	Perforated	3D printing digital light processing technology	NA	2–6 titanium pins	Shanghai Ruibo Medical Technology Co. Ltd.
Sumida et al. 2015	Clinical trial	NA	Geomagic^®^ Freeform^®^ (3D Systems, Rock Hill, SC, USA)	Pure Ti powder	0.5 mm and then laminated until 30 µm thick	Perforated with 1.0 mm-diameter pores	SLM-RP molding machine (selective laser melting method)	NA	2 screws	Eosint M 270^®^ (Electro Optical Systems, GmbH, Munich Germany)
Sagheb et al. 2017	Retrospective case study	Yxoss CBR^®^ backward by ReOss, Germany	CAD-CAM technology by ReOss Ltd. (Filderstadt, Germany)	Titanium powder	NA	Perforated	Selective laser melting: Ti powder melted layer by layer	Autoclaved at 135 °C for 20 min in confined water vapor	2 screws	Yxoss CBR^®^, (Filderstadt, Germany)
De Santis et al. 2022	Case series	NA	CAD/CAM software	(Class IV titanium)	NA	Perforated and needed to be covered with CM	Laser sintering CAD/CAM technology	NA. Came directly from the manufacturer.	5 mm long and 1.35 mm diam- eter mini titanium screws	Yxoss CBR^®^, ReOss, (Filderstadt, Germany)
Ghanaati et al. 2019	Case series	NA	CAM/CAM	(Class IV titanium)	NA	Perforated and needed to be covered with CM	NA	NA. Came directly from the manufacturer.	Fixed with screws and covered by CM	Yxoss CBR, ReOss, (Filderstadt, Germany)
Mandelli et al. 2021	Case series	NA	(DentalCAD, Exocad, GmbH, Germany)	1200 mpa zirconia	0.4–0.5 mm	Non-perforated	CAD/CAM miling	Autoclave (moist heat) with fractionated pre-vacuum at 134 °C, for 18 min. Drying time: 15 min.	Fixed with screws	Prettau, Zirkonzahn, South Tyrol Italy
Kim et al. 2024	Prospective RCT	Materialise Mimics (Materialise, Leuven, Belgium)	3-Matic (Materialise, Belgium)	Synthetic and alloplastic bone graft: mixture of hydroxyapatite and β-tricalcium phosphate in a 60:40 ratio with particle sizes 0.2–2.0 mm	Depending on the bone defect	Pore size between 0.7 and 1.2 mm; porosity 70–80%	Digital light processing and sintering at 1200 °C	NA	No fixation needed	OSTEON 3D (Dentium, Seoul, Republic of Korea)
Mekcha et al. 2023	Case series	Denti-Plan, NECTEC, Thailand	Geomagic Freeform^®^, 3D Systems, USA	3D-printed nanohydroxyapatite (3DHA)	Depending on the bone defect	Internal bone trabeculae: spherical macro-pores with a diameter of 1.75 mm and a thickness of 1.5 mm for the interconnected strut. The outer cortex: 2 mm thick. Cross-combined T-shaped (XT) patterns for internal structural reinforcement against collapse or damage during processing. The pore size of a graft ranged between 1.47 and 2.25 mm.	Layer by layer by a Projet160 3D printer, 300 × 450 dpi in resolution and 0.1 mm in layer thickness, using calcium sulfate hemihydrate in combination with a water-based binder	The printed grafts were soaked in a disodium hydrogen phosphate solution for 24 h to convert them into a hydroxyapatite structure, then dried and sterilized using ethylene oxide gas	No fixation screw was needed because 3DHA was completely fitted to the defect without any movement	The Touch™ Haptic Device, 3D System, SC. USA
Wang et al. 2023	RCT	Mimics software (20.0, Materialise, Leuven, Belgium)	NA	Freeze-dried allogenic bone	The pre-cut allogeneic bone block was taken from the iliac crest, and its dimensions were 20 × 15 × 12 cm	Customized allogeneic bone blocks (CABBs)	Milled from pre-cut cortico-cancellous freeze-dried allogeneic bone blocks	Cleaning, packaging, and sterilization not specified	Titanium pins: 8 mm long, 1.5 mm diameter	Osteolink Biomaterial Co. Ltd., Wuhan, China
Li S. et al. 2021	Retrospective clinical study	TRIOS 3 (3Shape, Copenhagen, Denmark)	Exocad, Darmstadt, Germany	Titanium without specifying	NA	Perforated	NA	Autoclave	Titanium screws	Biomet, FL, USA
Li L. et al. 2021	Retrospective case series study	Mimics Research software (Materialise, Leuven, Belgium)	3-Matic software (Materialise, Leuven, Belgium)	Titanium alloy powder (Dentarum Ti_6_Al_4_V, Germany)	0.3 mm reduced to nearly 0.2 mm	Perforated	3D printing machine laserCUSING^®^ (powder melting method)	Sterilized by high temperature and high pressure	2 or more screws	laserCUSING^®^ GmbH, lichtenfels Germany
Lizio et al. 2022	Pilot study	Mimics Innovation Suite, v17; Materialise, Belgium	CAD software Freeform Modelling Plus, version 13.0, 3D Systems	Ti64 powder	0.1–0.5 mm	Perforated	EOSINT M270 printer (Electro Optical Systems) and digital machine laser sintering (DMLS)	NA	2 or 3 screws	NA
Chiapasco et al. 2021	Retrospective clinical study	Yxoss CBR^®^ backward by ReOss, Germany	CAD-CAM technology by ReOss Ltd. (Filderstadt, Germany)	Class IV titanium	NA	Perforated	Laser sintering	NA	Titanium micro-screws	Yxoss CBR^®^ (Filderstadt, Germany)

**Table 2:** Overview of the benefits and limitations of customized titanium meshes used in GBR procedures.

Aspect	Advantages	Disadvantages
Defect adaptation	Patient-specific fit; prosthetically driven control of grafting materials distribution.	Longer design and production time compared to preformed meshes.
Mechanical support and space maintenance	Maintenance of the regenerative space; resistance to soft tissue compression.	Risk of exposure and dehiscence.
Production technology	High resolution and reproducibility; porous and complex designs.	High costs; sterilization and quality control required.
Biocompatibility	Excellent long-term stability and tissue integration.	Surface roughness may require additional finishing processes.
Sterilization	Compatible with both hot and cold sterilization methods.	Additional processing step if not provided by the manufacturer.
Clinical benefit	Reduces intraoperative time; minimal need for manual adaptation or fixation.	Not always readily available; design errors may lead to complications.

**Table 3:** Different available methods for customized mesh fabrication.

	Technique	Advantages	Disadvantages
Additive	Powder bed fusion - Selective laser melting (SLM) - Direct metal laser sintering (DMLS)	Can fabricate high-resolution porous structures. High shape flexibility. Short processing time. High accuracy and detail. Minimal fit discrepancy. Reduced material waste.	Post-processing is necessary to remove superficial partially melted particles. Tends to induce high tensile residual stresses. Support structures are needed for overhangs.
	Directed energy deposition (DED) - Electron beam melting (EBM)	Lower residual stresses in as-built parts. Excellent osseointegration and long-term biocompatibility in vivo.	Surface finishing is dependent on the material used. Post-processing finishing is needed to achieve the desired effect.
	Binder jetting	No support structures required. Can process materials that are challenging to melt (e.g., ceramics, composites). Unused powder can be reused. Wide range of materials. Fast process.	Lower mechanical performance compared to PBF. Limited success in producing metallic parts. Requirement for post-processing.
Subtractive	Five-axis CNC milling	Excellent dimensional precision and fit. Complex geometry capability. Wide range of materials available.	Difficult to fabricate complex or internal geometries. High material waste.
